# Amyotrophic lateral sclerosis caused by *TARDBP* mutations: from genetics to TDP-43 proteinopathy

**DOI:** 10.1016/S1474-4422(25)00109-7

**Published:** 2025-05-01

**Authors:** Rubika Balendra, Jemeen Sreedharan, Martina Hallegger, Raphaëlle Luisier, Hilal A Lashuel, Jenna M Gregory, Rickie Patani

**Affiliations:** Human Stem Cells and Neurodegeneration Laboratory, The Francis Crick Institute, London, UK; UK Dementia Research Institute at UCL, London, UK; Department of Basic and Clinical Neuroscience, Institute of Psychiatry, Psychology & Neuroscience, King’s College London, London, UK; UK Dementia Research Institute at King’s, London, UK; The Francis Crick Institute, London, UK; Oxford-GSK Institute of Molecular and Computational Medicine, Centre for Human Genetics, Nuffield Department of Medicine, University of Oxford, Oxford, UK; Genomics and Health Informatics Group, Idiap Research Institute, Martigny, Switzerland; Laboratory of Molecular and Chemical Biology of Neurodegeneration, Institute of Bioengineering, School of Life Sciences, Ecole Polytechnique Fédérale de Lausanne, Lausanne, Switzerland; Qatar Foundation, Doha, Qatar; Institute of Medical Sciences, University of Aberdeen, UK; Human Stem Cells and Neurodegeneration Laboratory, The Francis Crick Institute, London, UK; Department of Neuromuscular Disease, UCL Queen Square Institute of Neurology, London, UK

## Abstract

Mutations in the *TARDBP* gene, which encodes the TDP-43 protein, account for only 3–5% of familial cases of amyotrophic lateral sclerosis and less than 1% of cases that are apparently idiopathic. However, the discovery of neuronal inclusions of TDP-43 as the neuropathological hallmark in the majority of cases of amyotrophic lateral sclerosis has transformed our understanding of the pathomechanisms underlying neurodegeneration. An individual *TARDBP* mutation can cause phenotypic heterogeneity. Most mutations lie within the C-terminus of the TDP-43 protein. In pathological conditions, TDP-43 is mislocalised from the nucleus to the cytoplasm, where it can be phosphorylated, cleaved, and/or form insoluble aggregates. This mislocalisation leads to dysfunction of downstream pathways of RNA metabolism, proteostasis, mitochondrial function, oxidative stress, axonal transport, and local translation. Biomarkers for TDP-43 dysfunction and targeted therapies are being developed, justifying cautious optimism for personalised medicine approaches that could rescue the downstream effects of TDP-43 pathology.

## Introduction

Amyotrophic lateral sclerosis (ALS) is a progressive neurodegenerative disorder that renders patients paralysed and unable to eat, speak, and breathe.^1^ The cellular substrate of ALS is degeneration of upper and lower motor neurons, with glial cells and other cell types also being involved in the disease process. Approximately half of patients with ALS have cognitive impairment ^2^ and about 15% of patients will receive a diagnosis of frontotemporal dementia. ^1^ A small proportion (about 10%) of patients with ALS have a family history (familial ALS), while most patients are apparently idiopathic. ^1^ Approximately 40 genetic mutations have been associated with ALS,^3^ and most familial cases of the disease are due to autosomal dominant mutations, some of them with reduced penetrance. For the majority of patients with familial ALS and a substantial minority (approximately 10%) of patients with idiopathic ALS, there is an underlying causative gene mutation.^4,5^ Further genetic variants with smaller effect sizes contribute to ALS risk, with complex combinatorial effects.^6^

In this fourth paper of a Series on Genetic Amyotrophic Lateral Sclerosis,^7–9^ we review the evidence on *TARDBP* mutations that cause ALS and frontotemporal dementia. The *TARDBP* gene is located on chromosome 1 and encodes the protein TDP-43. Although *TARDBP* mutations are a rare cause of ALS, TDP-43 pathology is almost ubiquitous in patients with ALS and is also detected in a large proportion of patients with frontotemporal dementia. We also review the evidence linking TDP-43 pathology with ALS pathomechanisms, including the dysregulation of several aspects of cellular homoeostasis. Finally, we discuss translational aspects, focusing on biomarkers and the most promising avenues for therapeutic development.

## Genetics and epidemiology

Mutations in *TARDBP* account for 3–5% of cases of familial ALS and less than 1% of idiopathic ALS.^10,11^ More than 80 ALS-linked dominant mutations in *TARDBP* have been described in patients with ALS, but the specific pathomechanisms of these *TARDBP* variants remain unclear. It is striking that many of these variants affect the C-terminus of the TDP-43 protein, which has key functional roles and plays a central part in the formation of TDP-43 pathology.^12^ A small number of mutations also occur in the N-terminus, a domain that is necessary for TDP-43 to self-associate into oligomers that participate in RNA processing.^13^

Coding variants in *TARDBP* are extremely rare in healthy people, which is strongly suggestive of a causative pathogenic role for *TARDBP* mutations. One exception is Ala90Val, which is a variant within the nuclear localisation sequence of TDP-43 that has been described both in healthy control individuals and patients with ALS, suggesting that it might be a mutation with low penetrance.^10^ Although *TARDBP* mutations have been discovered across the world, there is a geographical hotspot (particularly of Ala382Thr) in Sardinia, Italy.^14^ Healthy *TARDBP* mutation carriers have also been described in people ages 65 years and younger in Sardinia, suggesting incomplete penetrance of Ala382Thr or additional protective genetic factors that might be co-inherited in this relatively isolated population.

Although the vast majority of *TARDBP* variants are missense mutations, two truncation mutations (Tyr374Ter and Trp385IlefsTer10)^15,16^ and a poorly characterised in-frame insertion-deletion mutation (Ser387delinsThrAsnPro)^17^ have also been described. The Ser387delinsThrAsnPro mutation was found in a patient with the flail arm variant of ALS^17^. The Tyr374Ter variant was found in a family with typical ALS. Analysis of skin-derived fibroblasts from patients has shown the expression of a truncated protein isoform. Furthermore, TDP-43 pathology was seen in spinal motor neurons in patient postmortem tissue. Interestingly, analysis of skin-derived fibroblasts from three patients with the Tyr374Ter variant showed a significant reduction in TDP-43 expression compared with expression in healthy people.^15^ The Trp385IlefsTer10 variant causes rimmed vacuole myopathy, but not ALS.

It is worth noting that a *TARDBP* 3′-untranslated region (UTR) variant that segregates with ALS has been found in two families.^18^ This variant lies within the TDP-43 binding region of the UTR, a region that is important for TDP-43 autoregulation. Specifically, this region contains UG motifs that are a target for TDP-43, which, upon binding, could trigger the splicing out of intron 7 within the UTR and alternative polyadenylation (a mechanism to regulate genes that leads to different 3′ ends to mRNAs) of the transcript. The consequence of this variant is an unstable transcript that is lost through mechanisms that remain unclear (possibly through nonsense-mediated decay).^19^ TDP-43 is able to regulate its own expression.^19^ Thus, it is important to establish whether genomic variation in the UTR of *TARDBP* causes disease by affecting TDP-43 expression. Although mechanistic studies to answer this question are outstanding, post-mortem analysis of the brain from a patient with the 3′ UTR variant showed elevated *TARDBP* mRNA expression, supporting the hypothesis that autoregulation is disturbed.^18^

## Clinical features

The phenotypic spectrum of pathogenic variants in *TARDBP* includes ALS (common), frontotemporal dementia (rare), mixed ALS and frontotemporal dementia (rare), and atypical neurological phenotypes (very rare; appendix 1 pages 4-13). The prognosis for *TARDBP*-ALS is variable and is associated with clinical features. The disease duration from symptom onset to death is 3–5 years.

Most variants cause typical ALS that is clinically indistinguishable from idiopathic presentations. However, an upper limb predominance at onset has been described.^20^ Within a family, an identical *TARDBP* mutation can cause clinically heterogeneous symptoms. For example, in the first family described with the Met337Val mutation, one male individual had bulbar onset, while his brother had limb onset.^10^ Given the rarity of *TARDBP* mutations, genotype-phenotype studies have been challenging, but some patterns are emerging. Intriguingly, Gly376Asp causes ALS, but Gly376Val was described to cause myopathy, with no evidence of neurodegeneration.^21^ The Trp385IlefsTer10 truncation is also associated with myopathy, specifically a rimmed vacuole myopathy, with TDP-43 aggregation observed in sarcomeres. In carriers of this mutation, neurogenic changes are mild and ALS or frontotemporal dementia have not been described.^16^ Rarer clinical phenotypes also associated with pathogenic variants include parkinsonism and REM sleep behaviour disorder.^22,23^ Some *TARDBP* variants can cause frontotemporal dementia (Pro112His and Ile383Val), and three lysine mutations are associated with mixed ALS and frontotemporal dementia (Lys176Ile, Lys181Glu, and Lys263Glu).^24–26^ The Asn267Ser and Ala382Thr mutations are also associated with mixed ALS and frontotemporal dementia.^27^ In carriers of Ala382Thr mutations, of the three frontotemporal dementia syndromes (behavioural variant, semantic variant primary progressive aphasia, and nonfluent variant primary progressive aphasia), the behavioural variant is the most common.^28^

## Neuropathology

**Figure 1:**
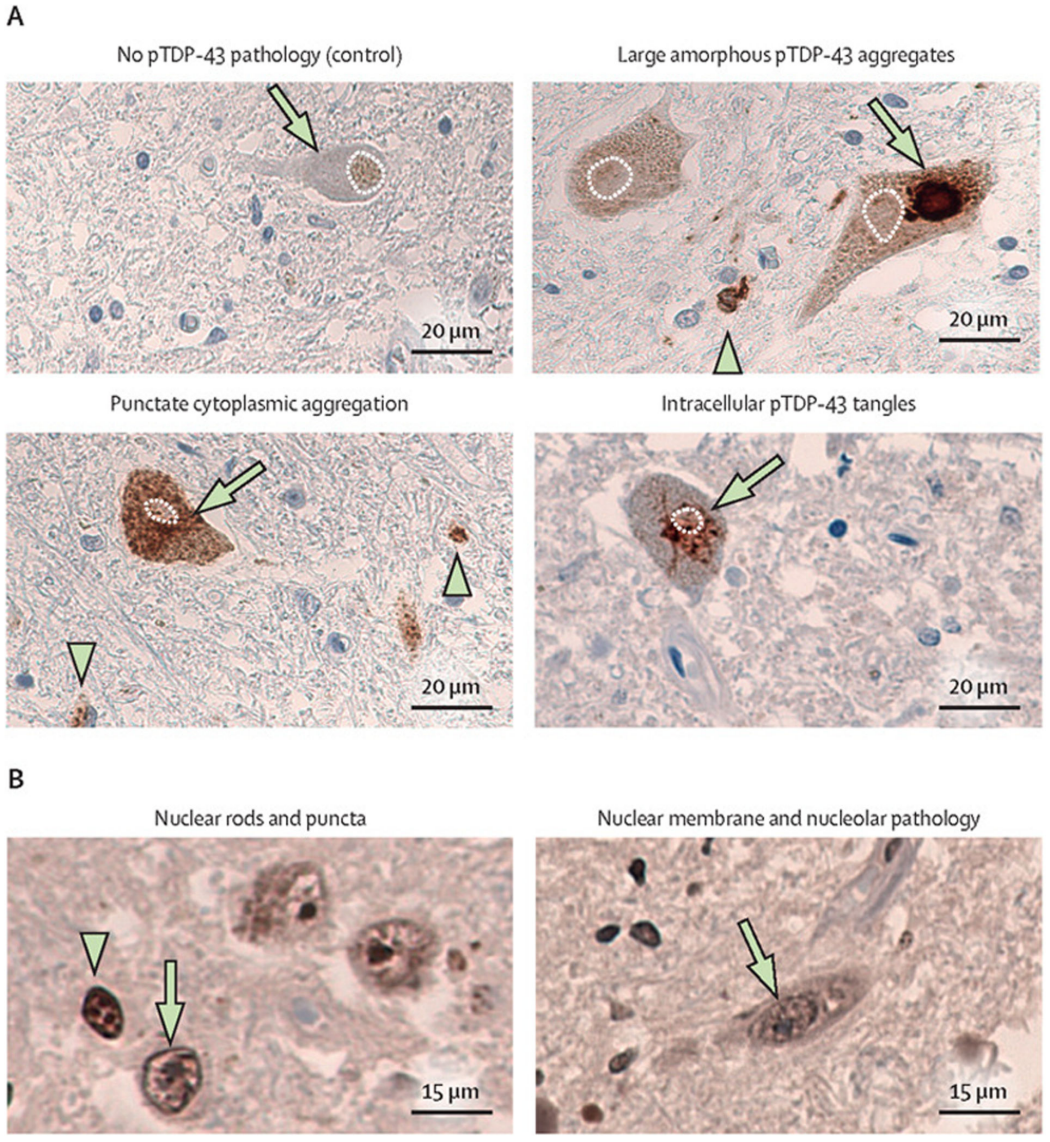
TDP-43 neuropathology in patients with amyotrophic lateral sclerosis (A) Morphologically distinct pTDP-43 aggregates. Representative photomicrographs (magnification 40x) from the anterior horn of the cervical spinal cord of patients with amyotrophic lateral sclerosis (as described by Spence et al^41^) and a healthy control individual. Neuronal TDP-43 pathology is heterogeneous and prominent glial pathology can be also detected. Green arrowheads indicate glia with TDP-43 pathology and white dotted lines show nuclear outlines. (B) Nuclear TDP-43^APT^ pathology. Representative photomicrographs (magnification 40x) from the anterior horn of the cervical spinal cord of patients with ALS (as described by Spence et al,^41^) showing that neuronal and glial nuclear pathology can be detected by use of sensitive techniques, such as RNA aptamers that stain pathological TDP-43 and can capture a wider range of aggregation than classical antibody staining, including nuclear aggregation.^41^ Green arrowheads indicate glia with TDP-43 pathology. TDP-43^APT^=RNA aptamers that stain pathological TDP-43.

Despite *TARDBP* mutations only accounting for about 3–5% of cases with familial ALS and less than 1% of those with idiopathic ALS, the TDP-43 protein is the major constituent of ubiquitinated cytoplasmic inclusions in the vast majority of people with ALS at postmortem, and in about 50% of people with frontotemporal dementia.^29^ The neuropathological features of *TARDBP*-ALS resemble those of TDP-43 pathology commonly detected in idiopathic patients with ALS or frontotemporal dementia (figure 1). TDP-43 is an RNA and DNA binding protein.^30,31^ It predominantly resides in the nucleus, although it can shuttle into the cytoplasm (panel 1), and plays key roles in pre-RNA splicing, polyadenylation selection, mRNA stabilisation, RNA transport, DNA repair mechanisms and biocondensate formation.^31–33, 45^ The key determinants of TDP-43 pathology formation are nuclear to cytoplasmic mislocalisation, although nuclear aggregates can also form; aberrant protein expression and aggregation; post-translational modifications (the process by which synthesised proteins are modified by the addition of chemicals); and impaired clearance of aggregates. TDP-43’s nuclear to cytoplasmic mislocalisation, phosphorylation, cleavage, and aggregation in the cytoplasm, with nuclear depletion, are hallmark features of ALS motor neurons.^34^

TDP-43 aggregates have a wide neuroanatomical distribution and heterogeneous cellular and morphological characteristics, but a distinct biochemical signature (such as distinct cleavage products and post-translational modifications). Different approaches to the histopathological classification of ALS and frontotemporal dementia have been proposed based on these features and include the specific cell type and subcellular localisation of TDP-43 pathology.^35,36^ At the morphological level, several (non-mutually exclusive) types of TDP-43 aggregates are commonly detected in neuropathological studies, including diffuse granular morphology; compact cytoplasmic inclusions; dot-like structures; thread-like structures; Pick-body-like structures; and granulofilamentous inclusions. Although progress has been made, our understanding of the biochemical, structural, and ultrastructural properties of TDP-43 aggregates and the relationship between TDP-43 neuropathological heterogeneity, clinical variability, and disease progression remains incomplete.

Brain-derived aggregates from different neuropathological subtypes of frontotemporal dementia have different seeding properties, spreading patterns, and induce the formation of different types of TDP-43 aggregates. However the relationship between these different types of TDP-43 aggregates with disease subtypes remains unclear.^37^ Furthermore, it is still uncertain whether different aggregate morphologies reflect different stages of TDP-43 pathology formation, maturation, or clearance, or if their distinct biochemical and morphological signatures reflect the existence of unique pathological strains or aggregation pathways. These differences might arise also from different cellular responses to modify or clear these aggregates.

Aggregates of TDP-43 contain a mixture of full-length and post-translationally modified forms of the protein. Substantial variations in the relative distribution of full-length, phosphorylated, and truncated forms of TDP-43 have been observed across neuropathological subtypes of frontotemporal dementia linked with TDP-43. The morphological diversity of TDP-43 aggregates might be influenced by differences in the biochemical composition and distribution of TDP-43. This diversity needs to be taken into consideration in therapeutic development to ensure targeting of all pathogenic forms of TDP-43. Notably, aggregate-based morphological classification alone is likely to be oversimplistic.^38^ Classification efforts have focused primarily on dystrophic neurites and neuronal cytoplasmic aggregates, excluding glial pathology. However, glial TDP-43 pathology is also frequently found,^39,40^ principally in oligodendrocytes and astrocytes.

The classic histopathological staging of TDP-43, in the context of ALS, is done using Braak staging.^40^ Braak staging classifies post-mortem cases into four categories according to the brain regions involved, and attributes the highest stage to that in which most brain regions have pathology. This staging is focused on the burden of TDP-43 in the brain as a whole and is reliant on the spreading of pathology from a primary locus, which remains controversial and has yet to be conclusively established for TDP-43 pathology. This staging system also does not consider the relative burden of pathology in each brain region, nor clinical heterogeneity. In a research context, TDP-43 pathology is instead graded using other means. Most commonly, TDP-43 is graded on a regional basis using an ordinal burden score (mild, moderate, or severe)^39^ or using digital burden scores, such as superpixel analysis.^41^ Indeed, even with sophisticated data-driven approaches to quantify TDP-43 pathology,^38^ misclassification can still occur, usually in the context of mixed pathological diagnoses, age-related changes, and in people with genetic mutations that lead to heterogeneous morphologies.

The presence of TDP-43 pathology in specific brain regions relates to the presence of clinical manifestations associated with that region.^39,40^ However, this relationship is not linear, as an increased burden of TDP-43 pathology does not result in increased symptom severity. Furthermore, TDP-43 pathology can be detected in individuals without ALS or frontotemporal dementia (e.g. in as many as 40% of people older than 65 years; panel 2).^42^ However, as tools to detect TDP-43 pathology become more sensitive compared to antibody-based methods, further insights have been gleaned. For example, the combined use of a method to detect TDP-43 loss of function using in-situ hybridisation probes targeting cryptic exons (an abnormality of RNA splicing leading to an intronic sequence being included in a mature mRNA) in conjunction with more sensitive (compared to antibodies) methods to detect pathological TDP-43 aggregates (RNA aptamers), suggest that TDP-43 pathology progresses from early nuclear to later cytoplasmic pathology.^41^

TDP-43 can separate into liquid-like compartments (liquid-liquid phase separation is a process in which biomolecules separate into liquid-like distinct compartments based on their biomolecular characteristics, enabling the formation of organelles without a membrane, such as nucleoli). This phase separation can be a stress-induced phenomenon in cellular models of the disease, for example, following arsenite treatment. This separation regulates many of TDP-43’s physiological functions, but could also lead to the transient inactivation of TDP-43, resulting in loss of interaction with its protein binding partners and interfering with TDP-43’s roles in splicing.^43^ Interestingly, differentially localised TDP-43 aggregates might have distinct origins: the liquid-liquid phase separation governs nuclear aggregates, whereas the formation of cytoplasmic inclusions seems to be aggresome-dependent (aggresomes are accumulations of misfolded proteins juxtaposed to the nucleus).^44^ Studies have reported the involvement of aggresome formation and liquid-liquid phase separation in TDP-43 cytoplasmic aggregation.^44^ These observations suggest that the formation of TDP-43 aggregates in different cellular compartments and cell types occurs through distinct and potentially competing pathways, thus giving rise to TDP-43 aggregates with different composition, dynamics, and toxic properties. This variability in aggregation mechanisms might contribute to the pathological heterogeneity observed in the brain, where different forms of TDP-43 aggregates could drive disease progression through cell type-specific mechanisms, which could ultimately influence disease phenotype, progression, and severity.^44^

## TDP-43 pathophysiology

*TARDBP*-ALS has proven difficult to generate a mouse model for because TDP-43 is highly dosage-sensitive, and any variation from physiological expression seems phenotypically consequential.^19,46^

**Figure 2:**
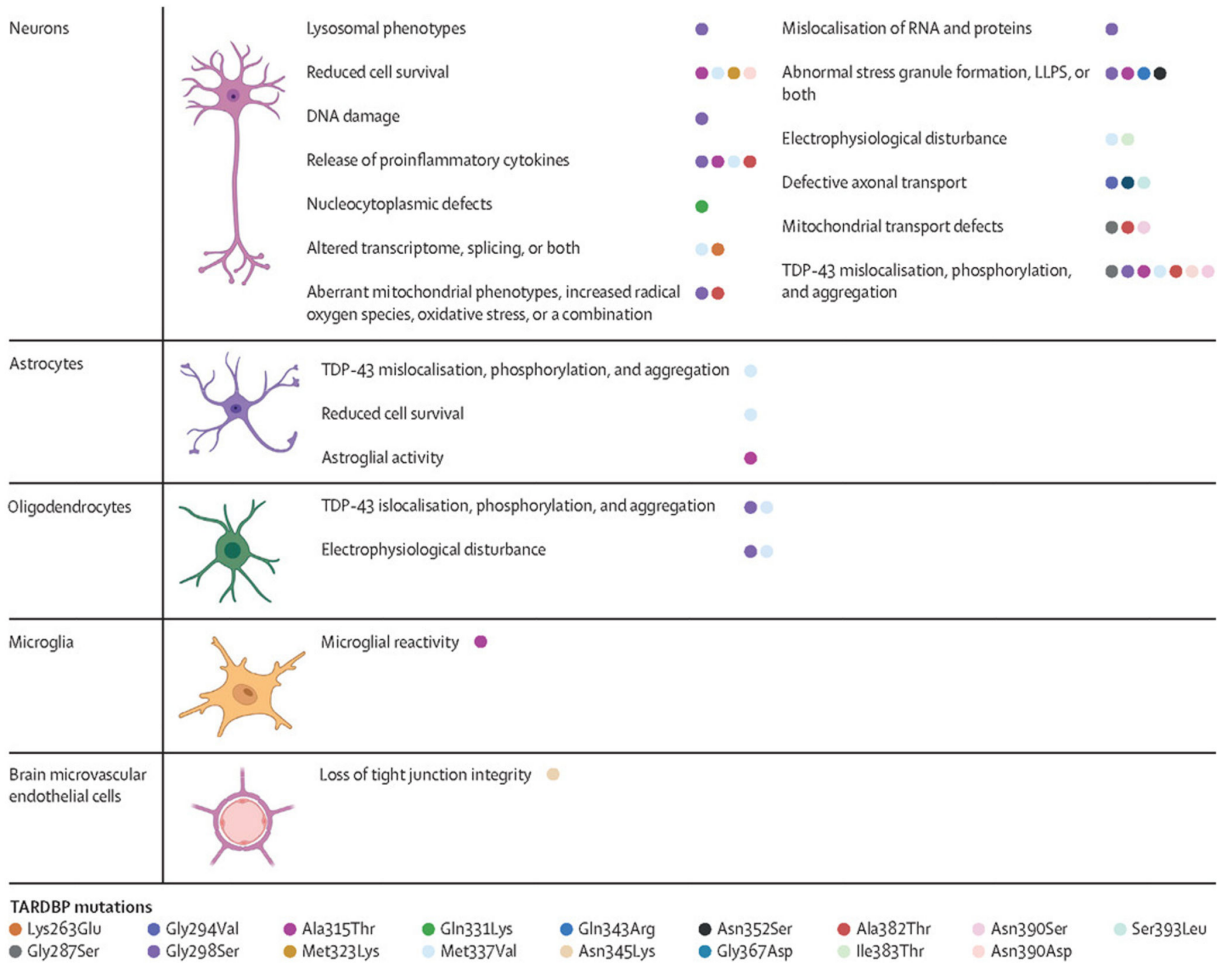
Cellular phenotypes caused by *TARDBP* mutations LLPS=liquid liquid phase separation. Originally created in BioRender. Balendra, R. et al. (2025) https://BioRender.com/l72h400.

The specific pathomechanisms of *TARDBP* variants remain unclear, with a range of cellular phenotypes identified in different cell types (figure 2; appendix pages 4-13). It is notable that around 70 of the 80 *TARDBP* mutations affect the C-terminal low complexity domain (a region of a protein that does not have the usual complexity of amino acids of a typical protein) of TDP-43, which has roles in protein–protein interactions and liquid-liquid phase separation.^12^ Despite detailed studies, mechanistic understanding about most *TARBDP* variants remains outstanding, and controversy remains as to how mutations might cause disease. In mice, the Gln331Lys mutation of the C-terminal domain has been found to disturb TDP-43 autoregulation, which results in an increase in TDP-43 expression and a subsequent gain of splicing function.^47^ These effects are in contrast with those of the Lys181Glu mutation, which is within RNA recognition motif (RRM) domain 1 and causes quite striking loss of RNA binding.^25,47^ Furthermore, variants in the 3′ UTR have been described and could disrupt the autoregulatory domain of *TARDBP*.^70^ A small number of mutations also occur in the N-terminus of TDP-43, a domain that is necessary for TDP-43 to self-associate into oligomers that can correctly function in RNA processing.^13^ Impaired TDP-43 dimerisation is also observed in brain tissue from patients with ALS, and expression of N-terminal dimerisation-impaired TDP-43 has been shown to cause TDP-43 pathology in vitro.^49^ These mutation-related effects are likely to be different when wild-type TDP-43 is mislocalised in non-*TARDBP* mutation related ALS and idiopathic ALS.

TDP-43 has an N-terminal domain (residues 1–76), two RNA recognition motifs (residues 106–176 (RRM1) and residues 191–259 (RRM2), and an intrinsically disordered C-terminal domain (residues 274–414), which contains multiple aggregation-prone motifs. Although the structure of the N-terminal domain and RRM domains of TDP-43 have been characterised by crystallography and nuclear magnetic resonance spectroscopy,^13,50^ the three-dimensional structure and conformational dynamics of the full-length recombinant protein remain incompletely resolved due to its high propensity to misfold and aggregate in vitro and the highly dynamic nature of its unstructured domains. Investigation has consequently prioritised TDP-43 fragments,^51^ but such emphasis might have constrained our understanding of how interdomain interactions influence TDP-43’s oligomerisation, post-translational modifications, interactome, and protein function.

Cryogenic-electron microscopy studies (a structural biology technique of flash freezing a molecule and studying its structure in a native state)^52,53^ have provided insight into the amyloid core structure of TDP-43 fibrils. The first structure was obtained using ex vivo samples from individuals with ALS.^52^ A subsequent study showed that the fibril fold derived from neuropathological FTLD-TDP type A (chevron fold) is distinct from that of FTLD-TDP type B (double-spiral fold).^54^ Interestingly, analysis of ex vivo fibrils from FTLD-TDP type C patient tissue revealed the formation of heteromeric fibrils of TDP-43 and annexin A11.^55^ Altogether, these experimental findings suggest that differences in the structural features and protein composition of the fibrils might be associated with different TDP-43 proteinopathies and could contribute to TDP-43 neuropathological heterogeneity. One limitation of these studies is that the structural features revealed are restricted to C-terminal domain residues that form the amyloid core of fibrils. The structure of fibrils derived from synthetic or recombinant C-terminal domain fragments differs substantially from that of the recombinant full-length TDP-43 or ex vivo TDP-43 fibrils,^51^ suggesting that the cellular milieu, differences in post-translational modifications, and interdomain cross-talk could be key determinants of the TDP-43 fibril core structure and its potential for co-aggregation with other proteins.^55^

Several post-translational modifications occur in the C-terminus, which also serves as a major interactome hub and regulator of TDP-43 subcellular distribution, liquid-liquid phase separation, and aggregation.^29,56^ Therefore, post-translational modifications within the C-terminus might act as molecular switches regulating its cellular properties, fibril structure, and response to stress conditions.^57^ Conversely, aberrant post-translational modifications could trigger the transition from native to pathological forms of the protein, thus contributing to disease initiation and progression (panel 3). It is noteworthy that our knowledge of the post-translational modifications of TDP-43 has been shaped by the focus on a few cleavage products and by the availability of antibodies, mainly against phosphorylated sites.

It has been proposed that TDP-43 aggregation contributes to its loss of function through multiple mechanisms, including sequestration of native TDP-43 into insoluble aggregates, nuclear depletion, disruption of protein–protein interactions, impairment of proteasomal degradation, and dysregulation of RNA splicing and processing.^58^ However, the C-terminal amyloid-forming domain is buried in the fibril core, while the structured N-terminal domain and the RNA recognition motifs are located at the surface of the aggregates. Therefore, TDP-43 fibrils might retain some physiological function or acquire new functions.^53^ These fibrils become seeding competent only after proteolytic cleavage of the structured domains, which exposes the amyloid core.

### Consequences of TDP-43 mislocalisation

Disruptions in TDP-43 function can profoundly impact RNA metabolism, including cryptic splice site usage,^59^ alternative polyadenylation,^60^ subcellular localisation,^61^ and stability.^62^ These disruptions have each been implicated in the pathogenesis of ALS and frontotemporal dementia. While TDP-43 binds to introns in pre-RNAs,^63^ aberrant mRNA metabolism is linked to its binding to RNA regulatory elements of the mRNA, including the 5' UTR, 3' UTR, and introns. TDP-43 binding to 3' UTRs is known to regulate mRNA stability and its interactions with 5' UTRs facilitate the transport of specific mRNAs to neurites.^64^ Non-coding regions of mRNAs, such as retained introns,^65^ can regulate the subcellular localisation of the RNA binding proteome.^66^ Additionally, TDP-43 has a role as repressor of cryptic exon usage and this role has revealed some specific functional targets of TDP-43,^67^ including stathmin-2 (*STMN2*)^68,69^ and unc-13 homolog A (*UNC13A*).^70,71^ Alternative splicing of TDP-43 could also contribute to the generation of modified forms of the protein. For instance, truncated isoforms that do not have the C-terminal domain but contain a putative nuclear export sequence were shown to be upregulated in motor neurons under conditions of hyperexcitability. This shorter, C-terminally truncated isoform alters the localisation of endogenous TDP-43 and contributes to the formation of inclusions.^72^

The versatile functions of TDP-43, shaped by its interactions with various RNA binding proteins, can explain why pinpointing a single pathomechanism remains challenging. Accumulating evidence suggests that TDP-43 exists in a dynamic equilibrium between different oligomeric states, stabilised by its interaction with RNAs and other proteins.^102^ An inappropriate stress response is a possible cause for protein aggregation, as several RNA-binding proteins associated with and linked to ALS, including TDP-43, are found in stress granules in response to several cellular stressors.^73^ But rather than being a driver of their formation, TDP-43 was reported to be recruited to stress granules.^74^ Furthermore, recombinantly formed amyloid-like fibrils can trigger cytoplasmic TDP-43 relocalisation and persistent cytoplasmic protein assemblies that are independent of conventional stress granules.^75^ By contrast, TDP-43 is involved in cell-specific regulation of stress granule dynamics.^74^ The cytoplasmic localisation of TDP-43 into stress granules and the high local concentration and proximity of TDP-43 molecules in these granules might turn the otherwise dynamic TDP-43 condensates into less dynamic, aged condensates, which then phase-transition into oligomers.

Most TDP-43 disease-causing mutations fall within its C-terminal domain in intrinsically disordered regions. These regions lack tertiary structure, have conformational flexibility, and allow proteins to concentrate into biomolecular condensates through liquid-liquid phase separation.^76^ Overlapping regions in the C-terminus of TDP-43 can cause its phase separation and aggregation, due to structural rearrangements.^52^ Stresses, such as proteotoxicity, mitochondrial dysfunction, and glutamate excitotoxicity might promote TDP-43 assembly, which could in turn induce misfolding and the formation of fibrillar aggregates. The physiological importance of TDP-43 condensation has been highlighted by experiments modulating its condensation potential through biophysically informed strategic mutations in intrinsically disordered regions, also in cells. ^45^ Condensation changes the binding to long RNA stretches and the regulation of these transcripts. A mechanism relevant to ALS could be explained by altered TDP-43 condensation and the consequential loss of autoregulatory binding to its own condensation-dependent binding site in the 3′ UTR, leading to an overexpression of TDP-43.^45^ Furthermore, ALS-linked mutations and TDP-43 isoforms modulating condensation can lead to defective anterograde and retrograde transport of TDP-43 ribonucleoprotein (a ribonucleoprotein (RNP) is a complex formed by the associated of RNA and RNA binding proteins; an RNP granule consists of RNA binding proteins and RNA that is formed through liquid-liquid phase separation), which could trigger motor neuron degeneration by impairing mRNA transport and local translation.^77^

Understanding the recruitment and release of TDP-43 from biomolecular condensates or its transition from liquid to solid state seems crucial for deciphering pathology. The inciting trigger of TDP-43 aggregation remains unknown. The formation of TDP-43 pathological aggregates likely reflects the failure of the quality control system to degrade misfolded TDP-43 and aggregated forms of the protein. Although the ubiquitin-proteasome system (a protein degradation system that targets a protein for breakdown through labelling with ubiquitin) and the autophagy lysosomal systems have been implicated in the clearance of TDP-43 aggregates, the specific roles and contributions of these pathways in clearing the different TDP-43 species and pathologies remain incompletely resolved.^78^ Several studies suggest that the ubiquitin-proteasome system is primarily involved in the clearance of native TDP-43, while the autophagy lysosomal system predominantly clears TDP-43 aggregates.^79^ Additionally, it has been shown that chaperone-mediated autophagy is involved in the turnover of both physiological and aggregated forms of TDP-43.^80^ Thus, dysregulation of autophagy, whether due to ageing, genetic predisposition, or lysosomal damage caused by TDP-43 aggregates, could be a major contributor to pathogenesis. Notably, TDP-43 has also been implicated in autophagy homoeostasis.^81^

TDP-43 mislocalisation has been recapitulated in human induced pluripotent stem cell (hiPSC)-derived spinal cord motor neurons and cortical neurons from mutation carriers of other ALS-associated genes Mislocalisation in the cytoplasm is associated with endoplasmic reticulum stress and impaired autophagy flux^82^ (appendix page 14). Furthermore, detergent-insoluble extracts from post-mortem samples of patients with idiopathic ALS have been transfected into hiPSC-derived motor neurons and astrocytes^83^ to investigate seeded aggregation. These studies revealed a prion-like spread (a process by which a misfolded and aggregated protein moves from one cell to another in a similar manner to the spread of prions) of TDP-43 in these cell culture models, leading to TDP-43 mislocalisation from the nucleus to the cytoplasm, its aggregation, and eventually cell death.

TDP-43 has been implicated in other pathomechanisms, including endoplasmic reticulum stress, mitochondrial dysfunction and oxidative stress (panel 4) and axonal transport and local translation (panel 5). Disrupted organellar function, for example endoplasmic reticulum stress, is both caused by, and itself drives, TDP-43 mislocalisation. The formation of TDP-43 aggregates impairs autophagy and disrupts cellular homoeostasis, thereby leading to an amplifying cycle that triggers downstream effects, that might ultimately lead to neurodegeneration.

### Effects in glial cells

Accumulating evidence suggests that glia regulate disease progression in ALS.^84^ In post-mortem tissue from patients with ALS, widespread glial TDP-43 aggregation has been described.^85^ Heterogeneity exists, with a small subset of idiopathic cases (around 7%) not having detectable glial pathology.^40^ Depleting TDP-43 from mature oligodendrocytes in mice leads to necroptosis of oligodendrocytes, shortened lifespan, and progressive motor phenotypes.^86^ In a zebrafish model, microglia can phagocytose TDP-43 in degenerating motor neurons; when microglia are depleted, TDP-43 is redistributed to the cytoplasm, the axons, and the extracellular space.^88^ In post-mortem tissue from patients with idiopathic ALS, higher TDP-43 burden correlated with increased microglial activation.^89^ A PET imaging study using the radioligand for activated microglia [^11^C]PK11195 of patients with familial frontotemporal dementia, including participants with mutations leading to TDP-43 pathology, showed increased microglial activation in frontotemporal regions.^90^ However, in a mouse model in which human TDP-43 pathology can be reversibly induced, only subtle microglial changes were found in the spinal cord, despite progressive motor neuron loss.^87^ In a study using detergent-insoluble extract from post-mortem tissue of patients with idiopathic ALS, motor neurons were more vulnerable than astrocytes to the seeded TDP-43 aggregation^83^

## Biomarkers for TDP-43 proteinopathy

Given the heterogeneity of TDP-43 pathology and the dysfunction of TDP-43 in ageing and in several neurodegenerative diseases, biomarkers of TDP-43 proteinopathy are warranted. A breadth of clinical and research indications ranging from diagnosis to prognostication and stratification in clinical trials, to target engagement and screening of mutation carriers stand to benefit from biomarker discovery. The accuracy of TDP-43 detection in CSF depends on the sensitivity of the techniques and is variable between studies.^91^ The detection of TDP-43 proteinopathy in blood samples would have wider utility and could be readily adapted for point of care testing. The use of plasma-derived extracellular vesicles (vesicles with a membrane that are released from cells into bodily fluids) is a promising approach. A study showed that the combination of extracellular vesicle-derived TDP-43 levels with 3-repeat or 4-repeat tau ratios had the ability to distinguish patients with ALS from those with frontotemporal dementia, as well as from healthy controls and people with other neurodegenerative diseases, such as Alzheimer’s disease.^92^ Other peripheral biomarkers in development include the detection of TDP-43 in platelets isolated from peripheral blood samples, which have disease-specific TDP-43 aggregation profiles and can carry neuronal and astrocyte-derived mRNA signatures. The detection of TDP-43 in platelets might therefore provide a window into TDP-43 pathology in the CNS.^93^ The isolation and examination of extracellular vesicles and platelets from blood samples is not standard practice in most clinical testing laboratories, making immediate translation challenging. However, these preliminary findings hold promise for future tests.

Studies identifying markers of TDP-43 loss of function are also emerging. Cryptic exons reflect abnormal RNA splicing, leading to the inclusion of an intronic sequence into a mature mRNA transcript.^67^ Cryptic exons resulting from TDP-43 loss of function can be detected in tissue from patients with ALS or frontotemporal dementia.^67,71,69,70,68,94^; however, the susceptibility of RNA to degradation might make the clinical translation of these data challenging. Instead, a more promising marker of loss of function might be the protein products of splicing dysfunction. Two studies have described the detection of these protein products in both blood^95^ and CSF^96^ of individuals with ALS or frontotemporal dementia. The first study described the development of a monoclonal antibody that detects a TDP-43-dependent epitope in the hepatoma-derived growth factor-related protein 2, encoded by the inclusion of a cryptic exon.^95^ Importantly, cryptic HDGF-related protein 2 can be detected using standard clinical laboratory techniques, such as enzyme-linked immunosorbent assay, in the blood of people with ALS or frontotemporal dementia, as well as in presymptomatic carriers of *C9orf72* mutations, highlighting the potential value of this biomarker in early detection and treatment trials in the future. The second study used a non-biased screening approach in CSF from people with ALS or frontotemporal dementia and found 18 de novo cryptic peptides across 13 genes.^96^ PET ligands for TDP-43 are being developed with the aim to track in vivo pathology,^97^ and will require validation before clinical use in human beings. Furthermore, seed amplification assays for TDP-43 are gaining traction, and they can have high sensitivity and specificity for TDP-43 detection in post-mortem tissue, CSF, and olfactory brushings.^98^

## Therapeutic approaches

**Figure 3:**
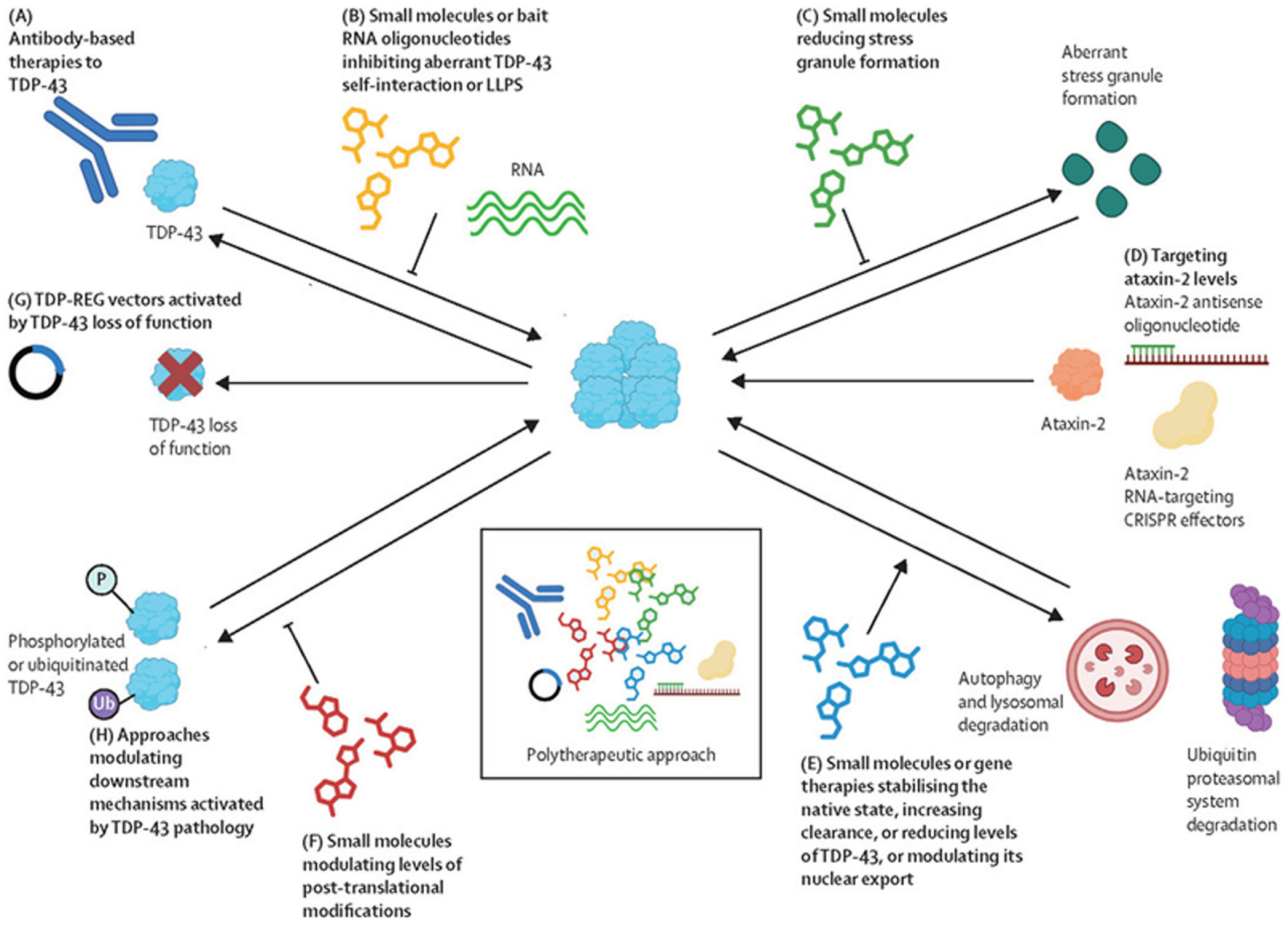
Therapeutic approaches for TDP-43 pathology A variety of approaches have been used in animal and in vitro models to target TDP-43. (A) Antibody-based therapies against TDP-43 have been investigated in cell and rodent models.^132–134,135^ (B) Small molecules, such as auranofin,^136^ chelethryne,^136^ or rTRD01 and nTRD22,^137,138^ or bait RNA oligonucleotides^101^ can inhibit TDP-43 self-interaction or aberrant phase transition in cell and in vivo models. (C) Small molecules can reduce stress granule formation in cell models, including those induced by TDP-43^139,140^ (D) Reducing the levels of ataxin-2 using antisense oligonucleotides^141^ or RNA-targeting CRISPR effectors^142^ ameliorates disease in cell and rodent models, but thus far, this strategy has not been clinically translated. (E) Small molecules can increase clearance of TDP-43^143,144^ or reduce levels of TDP-43,^145–148^, in some cases through an unknown mechanism, in cells and rodent models. A degron gene therapy vector (a protein sequence that can be recognised by E3 ubiquitin ligase, leading to protein degradation through the ubiquitin-proteasome system) also reduces TDP-43 levels and improves TDP-43 pathology in mouse disease models.^100^ Small molecules can also reduce the nuclear export of TDP-43.^107,149^ (F) Small molecules modulating levels of TDP-43 with post-translational modifications are effective in cell and rodent disease models^139,150,151^ and could be developed to target post-translational modifications directly. (G) Utilising TDP-43’s role in repressing cryptic exons, a TDP-REG therapeutic construct encoding a TDP-43/Raver1 fusion protein can be expressed only in diseased cells, including in a mouse model, leading to rescue of splicing changes.^99^ (H) Approaches to modulate downstream effects of TDP-43 pathology are being tested in a clinical trial (NCT05633459). LLPS=liquid-liquid phase separation. Originally created in BioRender. Balendra, R. et al (2025) https://BioRender.com/q62k493.

Alongside targeting TDP-43, the mechanisms affected by TDP-43 pathology detailed above provide potentially useful therapeutic targets (figure 3; appendix pages 4-13 and 15-31). Therapeutic developments include antibodies targeting TDP-43, small molecules that inhibit aberrant TDP-43 self-interaction or phase separation, and small molecules that increase TDP-43 clearance or its levels, or modulate the levels of TDP-43 with post-translational modifications. By exploiting TDP-43’s role in repressing cryptic exons, a study used a precision medicine gene therapy strategy that can rescue splicing changes specifically in cells with TDP-43 loss of function in a mouse model.^99^ In another study, exploiting the interaction of pathological isoforms of TDP-43 with the protein 14–3-3θ, a gene therapy strategy was able to reduce TDP-43 levels and improve TDP-43 pathology in mouse models.^100^ A polytherapeutic approach combining these different strategies could also be used. Strategies that directly target TDP-43 include bait RNA oligonucleotides, which reduce neurotoxicity in human cortical neuronal models of TDP-43 pathology.^101^ The most advanced of these therapeutic approaches are listed in the table.

Although growing evidence suggests that targeting post-translational modifications could be an effective strategy to inhibit TDP-43 aggregation, inclusion formation, seeding, and the spread of pathology, more studies are needed to decipher the role of different types of modifications and the cross-talk between them in regulating TDP-43 functions. Furthermore, a deeper understanding of which post-translational modifications are protective or pathogenic, and of how they influence TDP-43 properties or clearance at different stages of TDP-43 aggregation, inclusion formation, and spreading could pave the way for novel therapeutic strategies.

TDP-43 oligomerisation plays an important role in regulating its physiological functions, stability, and localisation,^13,44^ whereas misfolded TDP-43 oligomers act as intermediates on the pathway to TDP-43 fibrillisation and inclusion formation.^102^ However, very little is known about the biochemical (the post-translational modifications and their composition) and structural properties of physiological and misfolded oligomers, or the nature of TDP-43 oligomers. This knowledge gap has hindered the development of molecules and antibodies capable of differentiating between physiological and pathogenic TDP-43 oligomers, making it challenging to identify and selectively target the toxic oligomeric forms of the protein. This lack of knowledge, coupled with the heterogeneity of TDP-43 aggregates in the brain, poses significant challenges for the development of anti-aggregation therapies (e.g., antibodies or proteolysis targeting chimera [a method to degrade proteins by a ligand that binds to a protein of interest connected to a ligand for E3 ubiquitin ligase, leading to degradation through the ubiquitin-proteasome system]) that can selectively clear or neutralise pathogenic forms of TDP-43. Neutralisation in this context refers to inhibiting the toxicity of TDP-43 aggregates, by blocking their ability to seed and spread pathology, or altering their conformation to render them non-toxic.

Stabilising the native state of the protein instead might be an alternative strategy to inhibiting its misfolding and aggregation while preserving physiological functions.^103^ Studies have identified small molecule inhibitors and mutations that block TDP-43 fibrillisation while maintaining its ability to interact with its native ligands.^103^ Although this approach might be effective for early intervention, at advanced disease stages, seeding competent TDP-43 aggregates are already widespread in the brain and such a strategy might not be sufficient to stop the propagation of TDP-43 pathology. Therefore, combination therapy might be necessary that both prevents TDP-43 aggregation and promotes the clearance or neutralisation of existing aggregates to stop or delay the progression of the disease. The diverse functions and oligomerisation states of TDP-43 underscore the essential need for targeting and modulation of only pathogenic forms of the protein. Therefore, gaining a deeper understanding of the mechanisms responsible for the clearance of physiological and aggregated forms of TDP-43 is crucial for developing effective strategies to interfere with TDP-43 pathology and neurotoxicity.

TDP-43 aggregation occurs either through C-terminus assembly of misfolded TDP-43 monomers or oligomers, or via liquid-liquid phase separation into biomolecular condensates, where TDP-43 is ultimately converted into irreversible amyloid-like aggregates. Understanding the interplay between these different forms of TDP-43, their differential effects on cellular homoeostasis, and the molecular switches that regulate the dynamics of their formation, interconversion, and clearance is essential for developing safe and effective therapies.

## Conclusions and future directions

We have summarised the diverse mechanisms involved in TDP-43 proteinopathy. Understanding which processes are principally affected in which cell types and their likely non-cell autonomous consequences is of key importance in therapeutic design. Using a combination of model systems (e.g., hiPSCs and mouse models), alongside multimodal techniques with further validation in human post-mortem tissue is important to generate translational findings of clinical relevance.

It is noteworthy that, in carriers of *SOD1* or *FUS* mutations, TDP-43 pathology is rarely observed, but their cognate *SOD1* or *FUS* encoded proteinopathy abounds. Given that these pathologically divergent subtypes of ALS present with some overlapping clinical features, unifying mechanisms of disease might exist. For instance, some studies have identified molecular hallmarks across subtypes of ALS with or without TDP-43 proteinopathy.^65^ In this context, we propose some research priorities for the field (panel 6).

With advances in personalised medicine and after the establishment of a number of strategic funding initiatives and globally coordinated consortia, there is reason for cautious optimism that breakthrough discoveries and therapies will be soon on the horizon for patients with ALS.

## Supplementary Material

Supplementary table 1

## Figures and Tables

**Table:** Clinical trials of drugs with effects on TDP-43 or on the downstream consequences of TDP-43 pathology.[These do not all target TDP-43] ALS=amyotrophic lateral sclerosis. ALS-FRS(R)=Revised ALS Functional Rating Scale. hiPSC=human-induced pluripotent stem cell. MND-SMART: Motor Neuron Disease - Systematic Multi-arm Adaptive Randomised Trial.

	Mechanism	Type of trial	Participants	Study outcome measures	Status	ClinicalTrials.govID
QRL-201	Increases the expression of stathmin-2 (decreased stathmin-2 is a downstream of TDP-43 pathology)	Phase 1 multicentre, randomised, double-blind, placebo-controlled, multiple ascending dose study: 12-week treatment and up to 36-week monitoring	Idiopathic or *C9orf72* ALS	Safety/adverse events; pharmacokinetics	Recruiting	NCT05633459
Amantadine hydrochloride	Reduces TDP-43 aggregates in neurons in vitro	Phase 2/3 (MND SMART)	ALS	ALS-FRS(R) decline over 18 months; survival; cognition and behaviour; ALS stage; anxiety and depression; quality of life; safety/adverse events	Recruiting	NCT04302870
Ibudilast	Phosphodiesterase inhibitor; increases TDP-43 clearance in cell models and protects cells from TDP-43 induced cytotoxicity^144^	Phase 2b/3 multicentre, randomised, double-blind, placebo-controlled, parallel group study; 12 months and 6-month open-label extension	ALS	ALS-FRS(R) decline over 12 months; survival; muscle strength; quality of life; safety/adverse events	Recruiting	NCT04057898
Tideglusib	GSK-3 inhibitor; reduces phosphorylated TDP-43 levels and relocalises TDP-43 to nucleus in lymphoblasts; treatment of mouse model led to reduced TDP-43 phosphorylation in the spinal cord^151^	Phase 2	ALS	Adverse events; ALS-FRS(R) decline; respiratory function	Not yet recruiting	NCT05105958
Bosutnib	Src/c-Abl tyrosine kinase inhibitor; in a mouse model, this drug reduced TDP-43 levels, neuronal cell death in the brain and spinal cord, and reversed motor and cognitve phenotypes;^147^ it also restored synaptic proteins, astrocytic function, and neurotransmitter homoeostasis;^152^ the drug reduced TDP-43 levels and improved survival of hiPSC motor neurons carrying *TARBDP* mutatons^148^	Phase 1/2 open-label, multcentre trial	ALS	Safety/adverse events; ALS-FRS(R) decline; ALS severity; respiratory functon; muscle strength; blood neurofilaments; quality of life	Recruitng	NCT04744532

## Search strategy and selection criteria

References for this Series paper were identified by a search of PubMed with the search terms “*TARDBP*” and “TDP-43” from Jan 1, 2006, to Dec 15, 2024, restricted to articles in the English language. The final reference list was generated based on relevance to the topics covered in this paper.

## Panel 1 Nuclear and cytoplasmic functions of TDP-43

As an RNA regulator, TDP-43 binds to UG-rich RNA sequences within intronic regions or 3′-untranslated regions (UTRs) of a multitude of mRNAs, including the autoregulatory region in its own *TARDBP* transcript.^19,63^ TDP-43 has a nuclear localisation signal that mediates its active nuclear transport, whereas the protein can passively diffuse out of the nucleus to the cytoplasm.^104^ Nuclear RNA enhances TDP-43 nuclear localisation,^104,105^ but TDP-43 has also important cytoplasmic functions, including mRNA stability, transport, and localisation. The binding of TDP-43 to nuclear 3′ UTRs mediates 3′ end processing but, if maintained during nuclear egress, it will have implications on the cytoplasmic fate of the mRNA. For example, the binding of TDP-43 to the *NEFL* 3′ UTR plays a vital role in the transport of neurofilament into distal neuronal processes.^61^

The role of TDP-43 in nuclear RNA processing is well documented and has been linked to neuronal physiology.^67,68–70,72,99^ Therefore, a nuclear localisation of TDP-43 is crucial for neuronal health. The cytoplasmic localisation of TDP-43 can lead to new RNA binding and thereby cause additional toxicities.^64,106^ Indeed, TDP-43 proteinopathy functionally compromises factors regulating nucleocytoplasmic transport through sequestration and subcellular mislocalisation, leading to perturbation of nuclear protein import and RNA export.^107^ Restoration of nuclear import of TDP-43 by importin-α and karyopherin-β1 prevents and reverses TDP-43 fibrillisation and could be harnessed therapeutically to reinstate TDP-43 homoeostasis, thereby mitigating neurodegeneration.^108^

## Panel 2 TDP-43 proteinopathy in ageing and in other neurological diseases

Although TDP-43 is thought to be the hallmark pathology of amyotrophic lateral sclerosis and frontotemporal dementia, it has also been described in other motor neuron diseases, such as primary lateral sclerosis,^109^ as well as in other non-motor neurodegenerative diseases, including limbic-predominant age-related TDP-43 encephalopathy,^110^ Alzheimer’s disease,^111^ corticobasal degeneration,^112^ progressive supranuclear palsy,^113^ Huntington’s disease,^114^ Machado–Joseph disease,^115^ spinocerebellar ataxia type 2,^116^ and Lewy body dementia.^42^

Importantly, TDP-43 pathology is also a frequently observed neuropathological finding of ageing.^42^ A large post-mortem study examining 136 non-neurological control individuals older than 65 years showed that 40% of individuals had evidence of TDP-43 pathology.^42^ This same study also found TDP-43 pathology in about 72% (n=29) of people with Alzheimer’s disease and about 73% of brains with concomitant Lewy body dementia (n=11). These data raise the possibility that TDP-43 pathology could reflect a common molecular mechanism shared between many diseases, as well as ageing. There might be common genetic and environmental factors in other neurodegenerative diseases and in ageing that can converge in TDP-43 pathology. Case-controlled studies evaluating TDP-43 pathology as a diagnostic target or therapeutic biomarker will need to specifically account for the presence of age-related TDP-43 pathology.

## Panel 3 Post-translational modifications of TDP-43

Several post-translational modifications of TDP-43, including acetylation, SUMOylation, methylation, truncations, and phosphorylation have been identified. In brain samples from patients with amyotrophic lateral sclerosis, phosphorylation (e.g., on Ser409 or Ser410), truncations (caspase cleavage at Asp89 and Asp174 generates 25 kDa or 35 kDa C-terminal fragments), and ubiquitination are common. However, these modifications do not appear to be essential for TDP-43 aggregation in vitro, raising the possibility that they might occur post-aggregation or reflect a cellular response aimed at clearing TDP-43 aggregates.^72,73^ Studies of post-translational modifications have been driven by access to antibodies rather than a systematic unbiased and quantitative assessment of their distribution on native and aggregated forms of TDP-43 or identification of the enzymes that regulate specific modifications. Furthermore, post-translational modifications are often studied individually, frequently using TDP-43 fragments, even though multiple post-translational modifications can co-occur on the same TDP-43 molecule. Finally, despite the important roles of the C-terminal domain in driving TDP-43 aggregation, several studies have shown that mutations in the N-terminal domain or ligand binding in the RNA recognition motifs strongly inhibit TDP-43 aggregation and phase separation, suggesting complex interdomain interplay.^117^ These observations underscore the importance of mapping and investigating the effect of N-terminal modifications on TDP-43 structure, aggregation, and function.

## Panel 4 Mitochondrial dysfunction and oxidative stress

Mitochondrial dysfunction and oxidative stress have long been phenomena observed across experimental models and in post-mortem tissue studies.^118,119^ However, several drugs targeting these pathways, including edaravone and sodium phenylbutyrate-tauroursodeoxycholic acid have failed to produce robust translational progress. Although this failure has led many in the field to suggest that these pathways might not be therapeutically viable, there is evidence to suggest that a more nuanced approach might be beneficial. A study published in 2021 proposed that metal dyshomoeostasis together with mitochondrial dysfunction might be sufficient to either trigger or exacerbate progression of amyotrophic lateral sclerosis.^120^ Indeed, a preprint paper published in 2024 showed that ciclopirox olamine, a US Food and Drug Administration (FDA)-approved antifungal, can trigger TDP-43 pathology in mouse models and human cell lines, including in neurons derived from human induced pluripotent stem cell, as detected by the inclusion of TDP-43-associated cryptic exons.^121^ Cryptic exon inclusion (i.e. TDP-43 pathology) directly caused by ciclopirox olamine is driven by oxidative stress and heavy metal toxicity, suggesting that dysregulation of these pathways are important early-stage contributors to ALS pathophysiology, and that interventions to reverse or prevent these events in previous trials might have been delivered too late in the disease process, as these are likely to be early initiators of TDP-43 pathology. Indeed, clinical trial data that have led to the FDA approval of edaravone were based on post-hoc analyses showing efficacy in a subgroup of individuals in the early stages of the disease, thereby supporting this hypothesis.^122^ Mitochondrial dysfunction and oxidative stress might be early features in the disease process, and their therapeutic targeting might be better served by screening for early TDP-43 pathology using sensitive biomarkers, with interventions delivered before neuronal loss.

## Panel 5 Axonal transport and local translation

Motor neurons can extend axons up to 1 m long to their neuromuscular junctions, and the transport of organelles (mitochondria and lysosomes) and biomolecules (proteins, RNAs, and lipids) is essential for neuronal homoeostasis. An impairment of motor neuron axonal transport has been implicated in disease models of genetic amyotrophic lateral sclerosis, including in carriers of *TARDBP* mutations.^123^ TDP-43 regulates microtubule-dependent transport, which plays an important role in the subcellular spatial allocation of mRNAs.^61^ Indeed, local translation is crucial to the healthy function of a neuron. In samples from patients with amyotrophic lateral sclerosis, TDP-43 has been found to be hyperphosphorylated in axons and intramuscular nerve bundles^124^ and in motor neurons derived from human induced pluripotent stem cells from patients.^125^ Hyperphosphorylated TDP-43 leads to assembly of the Ras GTPase-activating protein-binding ribonucleoprotein G3BP1 condensates, and a resultant inhibition of local translation in distal axons and neuromuscular junctions.^125^ G3BP1 is involved in RNA transport. Furthermore, depleting TDP-43 from primary motor neurons leads to transcriptomic changes and reduced protein synthesis in axons.^126^ Aberrant dynamics of liquid-liquid phase separation disrupt TDP-43 function. These dynamics are also an important aspect of axonal pathogenesis. Ribonucleoprotein granules formed of mutant TDP-43 are more viscous compared to ribonucleoprotein granules formed of wild-type TDP-43 and cause aberrant dynamics of axonal transport in primary motor neurons.^127^ Together, these mechanisms might lead to disrupted mRNA axonal transport, release from ribonucleoprotein granules, and local translation. TDP-43 works together with survival motor neuron protein, Staufen and Fragile X messenger ribonucleoprotein 1, which are essential proteins involved in axonal transport of RNA.^125,128^ TDP-43 protein is also responsible for the transport of mRNAs in granules, including those encoding ribosomal proteins, which thereby permits axonal homoeostasis and local translation.^64^ *TARDBP* mutant human induced pluripotent stem cell lines showed defective mitochondrial and lysosomal axonal transport,^123,129^ which could be rescued by the inhibition of histone deacetylase 6.^123^ Decreased retrograde transport of endosomes has also been described in TDP-43^*Met337Val*^ mice.^130^ All this evidence from patients and a plethora of model systems highlights the importance of TDP-43 for motor neuronal homoeostasis, which could contribute to the selective vulnerability of these cells.

## Panel 6 Key unanswered questions and research priorities

- What are the mechanisms by which different *TARDBP* mutations cause neurological disease and how do these mutations cause diverse clinical phenotypes?
- Why is TDP-43 a common pathological entity both in familial and in idiopathic amyotrophic lateral sclerosis?
- Do *TARDBP* mutations predominantly lead to a gain or loss of function of TDP-43, or a combination of the two?
- How can the alterations in TDP-43 levels seen in human disease be recapitulated in experimental models?
- Which downstream mechanisms of TDP-43 dysfunction could be effective therapeutic targets for disease modulation?
- Which TDP-43 protein conformations (i.e., monomeric, oligomeric, or fibrillar species) are the key contributors to pathogenesis and what is the role of the different TDP-43 post-translational modifications?
- Which TDP-43 protein conformations (i.e., monomeric, oligomeric, or fibrillar species) should be targeted for therapeutic benefit?
- Of the therapies currently under development, which are most likely to lead to therapeutic benefits?
- Which TDP-43 biomarkers under development (e.g., extracellular vesicle-derived TDP-43, platelet-derived TDP-43, cryptic exons, and imaging-based and seed amplification assays) are most reliable for assessing disease onset, progression, and therapeutic response?
- Why is TDP-43 pathology detected in healthy individuals and what is the implication for the development of biomarkers and therapies?
- Beyond TDP-43, are other RNA binding proteins salient in the pathogenesis of familial and idiopathic amyotrophic lateral sclerosis?
