# Supplementary material for: Amyotrophic lateral sclerosis caused by TARDBP mutations: from genetics to TDP-43 proteinopathy
Source: Lancet Neurol. Author manuscript; Available in PMC 2025 May 17. (PMC7617675; doi:10.1016/S1474-4422(25)00109-7)
Supplement: Supplementary table 1 [file EMS205435-supplement-Supplementary_table_1.pdf]

THE LANCET
Neurology

Supplementary appendix

This appendix formed part of the original submission and has been peer reviewed. We post it as supplied by the authors.

Supplement to: Balendra R, Sreedharan J, Hallegger M, et al. Amyotrophic lateral sclerosis caused by TARDBP mutations: from genetics to TDP-43 proteinopathy. *Lancet Neurol* 2025; **24:** 456–470.

Supplementary Appendix

Amyotrophic lateral sclerosis caused by *TARDBP* mutations: from genetics to TDP-43 proteinopathy

Rubika Balendra, Jemeen Sreedharan, Martina Hallegger, Raphaëlle Luisier, Hilal A Lashuel, Jenna M Gregory, Rickie Patani

Human Stem Cells and Neurodegeneration Laboratory, The Francis Crick Institute, London, UK (R Balendra MRCP PhD, Prof R Patani FRCP PhD)**; UK Dementia Research Institute at UCL, London, UK** (R Balendra,)**; Department of Basic and Clinical Neuroscience, Institute of Psychiatry, Psychology & Neuroscience, King's College London, London, UK** (J Sreedharan MRCP PhD)**; UK Dementia Research Institute at King's, London, UK, The Francis Crick Institute, London, UK** (M Hallegger PhD)**; current address: Oxford-GSK Institute of Molecular and Computational Medicine, Centre for Human Genetics, Nuffield Department of Medicine, University of Oxford, Oxford, UK** (M Hallegger)**; Genomics and Health Informatics Group, Idiap Research Institute, Martigny, Switzerland** (R Luisier PhD)**; Laboratory of Molecular and Chemical Biology of Neurodegeneration, Institute of Bioengineering, School of Life Sciences, Ecole Polytechnique Fédérale de Lausanne, Lausanne, Switzerland** (Prof H A Lashuel PhD)**; Qatar Foundation, Doha, Qatar** (Prof H A Lashuel)**; Institute of Medical Sciences, University of Aberdeen, UK** (Prof J M Gregory MB PhD)**; Department of Neuromuscular Disease, UCL Queen Square Institute of Neurology, London, UK** (Prof R Patani)

Correspondence to:

Prof Rickie Patani, Department of Neuromuscular Disease, UCL Queen Square Institute of Neurology, London, UK, WC1N 3BG.

rickie.patani@ucl.ac.uk

or

Dr Rubika Balendra, UK Dementia Research Institute at UCL, London, UK, WC1E 6AE.

r.balendra@ucl.ac.uk

Supplementary Table 1

Selected *TARDBP* mutations identified by the resultant protein change. For each *TARDBP* mutation, clinical phenotypes are detailed. Evidence for involvement of downstream phenotypes from *TARDBP* mutant models for *in vitro* systems, human cells, hiPSC neurons or glial cells, rodent primary neurons and mouse models are detailed. Green text specifies *TARDBP* mutant models with evidence for therapeutic interventions. pp 4–13

Supplementary Table 2

hiPSC models of TDP-43 proteinopathy

This table details findings from hiPSC models examining TDP-43 proteinopathy. p 14

Supplementary Table 3

A multitude of approaches have been used to target TDP-43 in pre-clinical models. Approaches using antibodies, small molecules directly targeting TDP-43, small molecules targeting other pathways with effects on TDP-43, indirect and direct genetic approaches, peptides and bait RNA are detailed with the evidence for their efficacy in different disease models. pp 15–31

Supplementary Table 1

Predicted Protein Change	Clinical Phenotype and comments	*in vitro* (no cells)	Human/rodent cells	hiPSC neurons	hiPSC glia/other cells	Rodent primary neurons	*Drosophila* model	Mouse model
K176I	Mutated lysine residue associated with FTD phenotypes							
K181E	Mutated lysine residue associated with FTD phenotypes							
K263E	Mutated lysine residue associated with FTD phenotypes			Transcriptomic impairments; hiPSC-derived motor neurons displayed disrupted RNA processing (Imaizumi et al. 2022)				
N267S	Common variant							

G287S	Common variant			TDP-43 mislocalisation, phosphorylation, accumulated insoluble TDP-43 species containing high levels of C-terminal TDP-43 fragments and mitochondrial transport defects, improved with HDAC6 inhibition (Fazal et al 2021)				
G294V	Common variant			D-Sorbitol is known to induce stress granules, but treatment with this improves defective axonal transport (Kreiter et al. 2018)				
G295S	Common variant							

G298S	ALS founder variant in southern China			1. Purocymin-induced and TDP-43-associated stress granule formation; improved with small molecule with extended planar moeity (Fang et al. 2019) 2. Widespread mislocalisation of both RNAs and proteins between the nucleus and cytoplasm in addition to various cellular phenotypes including increased ROS generation, mitochondrial depolarisation, lysosomal phenotypes and DNA damage. Phenotypes rescued following treatment with a VCP D2 ATPase inhibitor (Harley et al. 2021; Ziff, Harley, et al. 2023). 3. cGAS/STING has been shown to drive NF-κB and interferon activation in TARDBP mutations and STING inhibition reduced death of hiPSCs (Yu et al. 2020) 4. Accumulation of insoluble TDP-43 fragments (Liu-	Oligodendrocytes: TDP-43 cytoplasmic inclusions and aberrant persistence of functional Ca2+-permeable AMPARs (Barton et al. 2021)		Rescue of larval locomotor function with small molecule acting on nuclear export pathway (Chou et al 2018)	Neuromuscular junction phenotypes and progress to spinal cord gliosis but in the absence of clear neurodegeneration - homozygous to a greater extent that heterozygous (Ebstein, Yagudayeva, and Shneider 2019).

				Yesucevitz et al. 2014) 5. Motor neurons - but not sensory or cortical neurons carrying the same mutations - captured up to one third of the transcriptomic changes seen in lower motor neurons microdissected from ALS postmortem tissue cases (Held et al. 2023) 6. Increase in axon initial segment (AIS) length, perturbed activity-dependent AIS plasticity and hyperexcitability (Harley et al. 2023)				

A315T	Common variant	An acridine derivative, AIM4, inhibits the in vitro phase separation of a TDP-43 C-terminal fragment with the TARDBPA315T mutation (Girdhar et al. 2020)				Reduced (TDP-43 related) granule density and mobility (Liu-Yesucevitz et al. 2014)		1. Repeated intrathecal TDP-43 monoclonal antibody treatment reduces TDP-43 mislocalisation and NF-κB activation (Pozzi et al 2020) 2. Treatment with a CK-1δ (protein casein kinase-1δ) inhibitor led to preserved motor neurons in the lumbar anterior horn, reduced TDP-43 phosphorylated and reduced microglial and astroglial reactivity (Martínez-González et al. 2020). 2. 3. cGAS/STING has been shown to drive NF-κB and interferon activation in TARDBP mutations and STING inhibition led to preservation of cortical neurons and improved motor performance (Yu et al. 2020).
M323K	Not found in humans, studied in a mouse model							Homozygous: Slowly progressive motor neuron death, aberrant splicing (Fratta et al. 2018)

Q331K			SecinH3, a cytohesin inhibitor, reduced toxicity induced by TDP-43Q331K overexpression in human cells by increasing autophagic flux (Hu et al. 2019) TDP-43Q331K overexpression in N2a cells led to cytoplasmic mislocalisation of Nup98 and cytoplasmic aggregates of Nup93, Nup107 and Nup214 (Chou et al. 2018)			Rescue of nucleocytoplasmic defects with small molecule acting on nuclear export pathway (Chou et al 2018)		Heterozygous mutation: disturbs TDP-43 autoregulation, increased TDP-43 expression, gain of splicing and cognitive dysfunction (White et al. 2018)

M337V	Common variant			1. Elevated levels of soluble and insoluble TDP-43 protein together with a survival phenotype and susceptibility to PI3K pathway (Bilican et al 2012) 2. Glutamate-induced calcium release and mitochondrial calcium buffering (Dafinca et al. 2020) 3. Electrophysiological aberrance (Devlin et al. 2015). 4. Increased cytosolic TDP-43, and this improved with an siRNA to TARDBP (Nishimura et al 2014) 5. Altered SORTILIN splicing and loss of activity-dependent BDNF secretion (Tann et al 2019), improved with CRISPR/Cas9 correction 6. cGAS/STING has been shown to drive NF-κB and interferon activation in TARDBP mutations and STING inhibition reduced death of hiPSCs (Yu et al. 2020).	1. Astrocytes: Increased TDP-43 expression, nucleocytoplasmic mislocalisation and a survival phenotype (Serio et al 2013) 2. Oligodendrocytes: TDP-43 cytoplasmic inclusions and aberrant persistence of functional Ca2+-permeable AMPARs (Barton et al. 2021)	Cumulative death rate improved with small molecule with extended planar moeity (Fang et al. 2019)		Neuromuscular junction phenotypes and progress to spinal cord gliosis but in the absence of clear neurodegeneration - homozygous to a greater extent that heterozygous (Ebstein, Yagudayeva, and Shneider 2019). Decreased retrograde transport of endosomes has been demonstrated (Sleigh et al. 2020).

Q343R				Elevated levels of arachidonic acid and, reduced by pharmacologically targeting this pathway with caffeic acid (5-LOX inhibitor) (Lee et al. 2021)		Reduced (TDP-43 related) granule density and mobility (Liu-Yesucevitz et al. 2014)		
N345K					hiPSCs differentiated to brain microvascular endothelial cells revealed cell autonomous increased permeability to small molecules due to loss of tight junction integrity (Matsuo et al. 2024)			
N352S				Purocymin-induced and TDP-43-associated stress granule formation; improved with small molecule with extended planar moeity (Fang et al. 2019)				
Y374Term	Atypical TDP-43 expression in person with ALS							

G376D				Dysregulated axonal growth (Mitsuzawa et al. 2021)				
A382T	Common variant in Sardinia leading to behavioural variant FTD with temporal lobe atrophy.			1. Specific vulnerability of hiPSC-derived motor but not sensory neurons to oxidative stress (Onda-Ohto et al. 2023) 2. TDP-43 mislocalisation, phosphorylation, accumulated insoluble TDP-43 species containing high levels of C-terminal TDP-43 fragments and mitochondrial transport defects, inproced with HDAC6 inhibition (Fazal et al 2021) 3. cGAS/STING has been shown to drive NF-κB and interferon activation in TARDBP mutations and STING inhibition reduced death of hiPSCs (Yu et al. 2020).				
I383T				Glutamate-induced calcium release and mitochondrial calcium buffering (Dafinca et al. 2020)				

N390S				TDP-43 mislocalisation, phosphorylation, accumulated insoluble TDP-43 species containing high levels of C-terminal TDP-43 fragments and mitochondrial transport defects, improved with HDAC6 inhibition (Fazal et al 2021)				
N390D								Heterozygous: TDP-43 pathology and motor neuron degeneration (Huang et al 2020)
S393L				D-Sorbitol is known to induce stress granules, but treatment with this improves defective axonal transport (Kreiter et al 2018)				

Supplementary Table 2

Reference	Findings
Hall et al. 2017	Wild-type TDP-43 mislocalisation in VCP-mutant hiPSC-derived spinal cord motor neurons coincide with ER stress.
Smethurst et al. 2020	Sarkosyl insoluble extracts from sporadic ALS post-mortem tissue transfected into control hiPSC-derived motor neurons and astrocytes to study seeded aggregation. Demonstrated a prion-like spread of TDP-43 in these cell culture models, leading to TDP-43 mislocalisation from the nucleus to the cytoplasm, aggregation and cell death. Motor neurons were more vulnerable than astrocytes to the seeded aggregation.
Weskamp et al. 2020	Hyperexcitability leads to alternative splicing of *TARDBP* and to shortened TDP-43 (lacking a C-terminus) accumulating in cytoplasm in hiPSC-neurons.
Altman et al. 2021	TDP-43 has been found to accumulate in axons in hiPSC derived motor neurons from ALS patients with *C9orf72* mutations, leading to assembly of G3BP1 ribonucleoprotein condensates and a resultant inhibition of local translation in distal axons and NMJs
Ziff, Neeves, et al. 2023	All public data from ALS hiPSC-derived motor neurons used (429 hiPSC-MNs) and demonstrated a positive correlation between TDP-43 nuclear depletion and p53 activation, in the context of demonstrating that genome instability is a hallmark of sALS and fALS.
Hung and Patani 2024	Wild-type TDP-43 mislocalisation in VCP-mutant hiPSC-derived cortical neurons.

Supplementary Table 3

Type of therapy	Molecule	Compound type / Mechanism of action	*in vitro* (no cells)	Yeast	Rodent cells lines / primary neurons	Human cells	hiPSCs	*Drosophila in vivo* model	Rodent *in vivo* model
**Antibody**	A single-chain variable fragment intrabody derived from a monoclonal antibody to TDP-43	Bind to TDP-43 protein			Improves viability in HEK293A cells and N2a cells through proteasome, autophagy-lysosomal pathways and HSP70 (Tamaki et al. 2018)	Reduced mutant TDP-43 aggregation in HEK293A cells and improves viability in HEK293A cells through proteasome, autophagy-lysosomal pathways and HSP70 (Tamaki et al. 2018)			Reduced TDP-43 aggregation in embryonic mouse brain overexpressing mutant TDP-43 (Tamaki et al. 2018)

	Monoclonal antibody targeting C-erminal domain of TDP-43	Bind to TDP-43 protein	Reduces pathogenic TDP-43 seed amplification in CSF from sporadic ALS patients (Audrain et al. 2023)			Reduced seeding induced by FTD-TDP brain derived extracts in vitro (Afroz et al. 2023)			Reduced aggregrated and pheophorylated TDP-43 in rNLS8 mouse model and reduced neuronal loss resulting from inoculating FTD-TDP brain extracts in a mouse model (Afroz et al. 2023)
**Small molecules**	Auranofin	Organic gold thiol compound			Inhibits TDP-43 self-interaction in a screen of compounds in mouse cell lines (Oberstadt et al. 2018)				

	Chelethryne	Benzophenanthridine alkaloid			Inhibits TDP-43 self-interaction in a screen of compounds in mouse cell lines (Oberstadt et al. 2018)				
	N-acetylcysteine	Antioxidant				Reduced stress granule formation, TDP-43 ubiquitylation and insolubility in a human cell model (Hans, Glasebach, and Kahle 2020)			

	Protein casein kinase-1δ (CK-1δ) inhibitor	Protein casein kinase-1δ (CK-1δ) inhibitor				Decreased the phosphorylation of TDP-43, and improved its nucleo-cytoplasmic mislocalisation in lymphoblasts from sporadic ALS patients (Posa et al. 2019)			
	Small molecules inhibiting CK1	CK1 inhibition				Led to lower levels of TDP-43 inclusions in a neuronal cell model (Hicks et al. 2020).			

	rTRD01 and nTRD22: Compounds binding to TDP-43's RRMs or N terminal domain (in silico screen)	Bind to TDP-43, modulate TDP-43-RNA interaction			Reduced TDP-43 levels in primary motor neurons (Mollasalehi et al. 2020).			Small molecules which improved the locomotor function of Drosophila larval models of ALS (François-Moutal et al. 2019; Mollasalehi et al. 2020).	
	Trimethylamine N-oxide	Chemical chaperone	Increases the LLPS of TDP-43, whilst inhibiting its fibrillation (Choi et al. 2018)						

	Compounds which inhibit TDP-43 nuclear export	Nuclear export inhibition			Improvements of survival of primary motor neurons overexpressing wild type TDP-43 (Archbold et al. 2018)				Partial rescue of a motor phenotype in a TDP-43 overexpression rat model (Archbold et al. 2018).
	VCP D2 ATPase inhibitor						Rescue molecular and cellular phenotypes in both *TARDBP* and *VCP* mutations in hiPSC-derived motor neurons (Ziff, Harley, et al. 2023).		

	PARP inhibitors	PARP inhibition			Rescued primary neuronal cultures from TDP-43 mediated toxicity (L. McGurk et al. 2018)	1. Reduced arsenite-induced TDP-43 aggregation in human cells (L. McGurk et al. 2018) 2. Reduced TDP-43 induced cytotoxicity in a neural cell model (Duan et al. 2019)			
	Small molecule inhibitor of Tankyrase-1/2 (part of the PARP family)	Tankyrase 1/2 inhibition				Reduced arsenite-induced TDP-43 cytoplasmic foci in a human cell line (Leeanne McGurk et al. 2018).			

	Diallyl trisulfide	Organosulphur compound				Clearance of overexpressed TDP-43 by inducing autophagy (Liu et al. 2018)			
	EN6	Autophagy activator				Clearance of overexpressed TDP-43 by inducing autophagy (C. Y.-S. Chung et al. 2019)			
	MEK5 inhibition	MEK5 is a component of the autophagy-lysosome pathway				Reduced TDP-43 in the cytoplasm and toxicity in neural cells overexpressing TDP-43 (Jo et al. 2019)			

	Trehalose	Autophagy activator				Led to decreased TDP-43 levels in human cells overexpressing TDP-43 (Y. Wang et al. 2018).			
	AIM4 (acridine derivative)	Reduce TDP-43 aggregation		Inhibits TDP-43 aggregation in yeast model (Prasad et al. 2016)					
	Ibudilast - in clinical trial (See Table 1)	Phosphodiesterase inhibitor, enhances autophagy				Reduced TDP-43 aggregates in transfected HEK cells and rescued survival in NSC-34 cells overexpressing TDP-43 (Chen et al 2020)			

	Tideglusib - in clinical trial (See Table 1)	Non-ATP competitive GSK-3β inhibitor			Reduced pTDP-43 levels and improved TDP-43 nuclear localisation in a TDP-43 neuroblastoma model (Martínez-González et al. 2021).	Reduced pTDP-43 levels and improved TDP-43 nuclear localisation in ALS lymphoblasts (Martínez-González et al. 2021).			Oral treatment reduced pTDP-43 in the spinal cord of Prp-hTDP-43A315T mouse model (Martínez-González et al. 2021).

	Bosutinib - in clinical trial (See Table 1)	Src/c-Abl inhibitor, tyrosine kinase inhibitor					Reduced fragmented/misfolded TDP-43 and increased survival in *TARDBP* mutant hiPSC lines (Imamura et al. 2017).		Reduced TDP-43 postive cells in mouse TDP-43 model, reduced neuronal cell death in brain and spinal cord and reversed motor and cognitive phenotypes (Wenqiang et al. 2014). Restored synpatic proteins, astrocytic function and neurotransmitter homeostasis in TDP-43 mice (Heyburn et al. 2016).

**Bait RNA**	Bait RNA oligonucleotides which bind to TDP-43	Bind to TDP-43				Improve aberrant TDP-43 phase transition in TDP-43 overexpressing human cortical neurons, and can reduce neurotoxicity (Mann et al. 2019)			

**Indirect genetic approaches**	*Ataxin 2* ASO	Reduced *Ataxin 2* mRNA							Leads to improved phenotypes in a mouse model overexpressing human TDP-43 (Becker et al. 2017) Note that Biogen/Ionis Phase 1/2 clinical trial terminated due to no reduction in NfL (https://investors.biogen.com/news-releases/news-release-details/biogen-and-ionis-announce-topline-phase-12-study-results)

	Ataxin 2 targeting with RNA-targeting CRISPR effector proteins					RfxCas13d reduced TDP-43 aggregation in human cells (Zeballos et al. 2023)			RfxCas13d improved motor phenotypes, slowed disease progression and reduced TDP-43 in a mouse TDP-43 model (Zeballos et al. 2023)

	CHMP7 ASO	Reduced *CHMP7* mRNA					Leads to an improved disease phenotype and TDP-43-related molecular changes, and TDP-43 mislocalisation in a subset of sporadic ALS hiPSC lines (Coyne et al. 2021).		
	Degron gene therapy vector targeting interaction of 14-3-3θ with TDP-43	Degrades TDP-43							Improved phenotypes in three mouse models of disease (Ke et al. 2024)

**Genetic approaches targeting TDP-43**	TDP-REG vectors encoding a TDP-43/Raver1 fusion protein	Activated in cells with TDP loss of function				TDP-REG is activated by TDP-43 loss of function and leads to autoregulated splicing rescue in human cells (Wilkins et al. 2024)			TDP-REG is activated by TDP-43 loss of function in mice (Wilkins et al. 2024)
**Peptides**	D4 peptide - hydrophobic motif, TDP recognition motif and cell penetrating peptide motif				Reduces TDP-43 levels in N2A cells overexpressing TDP-43 (Gao et al. 2019)				Reduces TDP-43 levels in *Drosophila* overexpressing TDP-43 in muscles (Gao et al. 2019)
	QBP1 (small peptide)	Polyglutamine amyloid inhibitor	Binds TDP-43 and prevent its amyloid formation *in vitro*						

			(Mompeán et al. 2019).						

Supplementary Tables - References

1 Bilican B, Serio A, Barmada SJ, *et al.* Mutant induced pluripotent stem cell lines recapitulate aspects of TDP-43 proteinopathies and reveal cell-specific vulnerability. *Proc Natl Acad Sci U S A* 2012; **109**: 5803–8.

2 Manohar V, Crowley L, Sreedharan J. -Related Amyotrophic Lateral Sclerosis-Frontotemporal Dementia. In: Adam MP, Feldman J, Mirzaa GM, Pagon RA, Wallace SE, Amemiya A, eds. GeneReviews. Seattle (WA): University of Washington, Seattle, 2009.

3 Held A, Adler M, Marques C, *et al.* iPSC motor neurons, but not other derived cell types, capture gene expression changes in postmortem sporadic ALS motor neurons. *Cell Rep* 2023; **42**: 113046.

4 Lee H, Lee JJ, Park NY, *et al.* Multi-omic analysis of selectively vulnerable motor neuron subtypes implicates altered lipid metabolism in ALS. *Nat Neurosci* 2021; **24**: 1673–85.

5 Moujalled D, Grubman A, Acevedo K, *et al.* TDP-43 mutations causing amyotrophic lateral sclerosis are associated with altered expression of RNA-binding protein hnRNP K and affect the Nrf2 antioxidant pathway. *Hum Mol Genet* 2017; **26**: 1732–46.

6 Mitsuzawa S, Suzuki N, Akiyama T, *et al.* Reduced PHOX2B stability causes axonal growth impairment in motor neurons with TARDBP mutations. *Stem Cell Reports* 2021; **16**: 1527–41.

7 Onda-Ohto A, Hasegawa-Ogawa M, Matsuno H, *et al.* Specific vulnerability of iPSC-derived motor neurons with TDP-43 gene mutation to oxidative stress. *Mol Brain* 2023; **16**: 62.

8 Dafinca R, Barbagallo P, Farrimond L, *et al.* Impairment of Mitochondrial Calcium Buffering Links Mutations in C9ORF72 and TARDBP in iPS-Derived Motor Neurons from Patients with ALS/FTD. *Stem Cell Reports* 2020; **14**: 892–908.

9 Devlin A-C, Burr K, Borooah S, *et al.* Human iPSC-derived motoneurons harbouring TARDBP or C9ORF72 ALS mutations are dysfunctional despite maintaining viability. *Nat Commun* 2015; **6**: 5999.

10 Harley P, Kerins C, Gatt A, *et al.* Aberrant axon initial segment plasticity and intrinsic excitability of ALS hiPSC motor neurons. *Cell Rep* 2023; **42**: 113509.

11 Liu-Yesucevitz L, Lin AY, Ebata A, *et al.* ALS-linked mutations enlarge TDP-43-enriched neuronal RNA granules in the dendritic arbor. *J Neurosci* 2014; **34**: 4167–74.

12 Serio A, Bilican B, Barmada SJ, *et al.* Astrocyte pathology and the absence of non-cell autonomy in an induced pluripotent stem cell model of TDP-43 proteinopathy. *Proc Natl Acad Sci U S A* 2013; **110**: 4697–702.

13 Barton SK, Magnani D, James OG, *et al.* Transactive response DNA-binding protein-43 proteinopathy in oligodendrocytes revealed using an induced pluripotent stem cell model. *Brain Commun* 2021; **3**: fcab255.

14 Matsuo K, Nagamatsu J, Nagata K, *et al.* Establishment of a novel amyotrophic lateral sclerosis patient ( )-derived brain microvascular endothelial cell model reveals defective Wnt/β-catenin signaling: investigating diffusion barrier dysfunction and immune cell interaction. *Front Cell Dev Biol* 2024; **12**: 1357204.

15 Fang MY, Markmiller S, Vu AQ, *et al.* Small-Molecule Modulation of TDP-43 Recruitment to Stress Granules Prevents Persistent TDP-43 Accumulation in ALS/FTD. *Neuron* 2019; **103**: 802–19.e11.

16 Pozzi S, Codron P, Soucy G, *et al.* Monoclonal full-length antibody against TAR DNA binding protein 43 reduces related proteinopathy in neurons. *JCI Insight* 2020; **5**. DOI:10.1172/jci.insight.140420.

17 Yu C-H, Davidson S, Harapas CR, *et al.* TDP-43 Triggers Mitochondrial DNA Release via mPTP to Activate cGAS/STING in ALS. *Cell* 2020; **183**: 636–49.e18.

18 Kreiter N, Pal A, Lojewski X, *et al.* Age-dependent neurodegeneration and organelle transport deficiencies in mutant TDP43 patient-derived neurons are independent of TDP43 aggregation. *Neurobiol Dis* 2018; **115**: 167–81.

19 Martínez-González L, Rodríguez-Cueto C, Cabezudo D, *et al.* Motor neuron preservation and decrease of in vivo TDP-43 phosphorylation by protein CK-1δ kinase inhibitor treatment. *Sci Rep* 2020; **10**: 4449.

20 Fazal R, Boeynaems S, Swijsen A, *et al.* HDAC6 inhibition restores TDP-43 pathology and axonal transport defects in human motor neurons with TARDBP mutations. *EMBO J* 2021; **40**: e106177.

21 Harley J, Hagemann C, Serio A, Patani R. TDP-43 and FUS mislocalization in VCP mutant motor neurons is reversed by pharmacological inhibition of the VCP D2 ATPase domain. *Brain Commun* 2021; **3**: fcab166.

22 Ziff OJ, Harley J, Wang Y, *et al.* Nucleocytoplasmic mRNA redistribution accompanies RNA binding protein mislocalization in ALS motor neurons and is restored by VCP ATPase inhibition. *Neuron* 2023; **111**: 3011–27.e7.

23 Imaizumi K, Ideno H, Sato T, Morimoto S, Okano H. Pathogenic mutation of TDP-43 impairs RNA processing in a cell type-specific manner: Implications for the pathogenesis of ALS/FTLD. *eNeuro* 2022; **9**: ENEURO.0061–22.2022.

24 Chou C-C, Zhang Y, Umoh ME, *et al.* TDP-43 pathology disrupts nuclear pore complexes and nucleocytoplasmic transport in ALS/FTD. *Nat Neurosci* 2018; **21**: 228–39.

25 Ebstein SY, Yagudayeva I, Shneider NA. Mutant TDP-43 Causes Early-Stage Dose-Dependent Motor Neuron Degeneration in a TARDBP Knockin Mouse Model of ALS.

Cell Rep 2019; **26**: 364–73.e4.

26 Girdhar A, Bharathi V, Tiwari VR, *et al.* Computational insights into mechanism of AIM4-mediated inhibition of aggregation of TDP-43 protein implicated in ALS and evidence for in vitro inhibition of liquid-liquid phase separation (LLPS) of TDP-43-A315T by AIM4. *Int J Biol Macromol* 2020; **147**: 117–30.

27 Fratta P, Sivakumar P, Humphrey J, *et al.* Mice with endogenous TDP-43 mutations exhibit gain of splicing function and characteristics of amyotrophic lateral sclerosis. *EMBO J* 2018; **37**. DOI:10.15252/embj.201798684.

28 Hu W, Liu X, Wang S, Sun G, Zhao R, Lu H. SecinH3 attenuates TDP-43 p.Q331K-induced neuronal toxicity by suppressing endoplasmic reticulum stress and enhancing autophagic flux. *IUBMB Life* 2019; **71**: 192–9.

29 White MA, Kim E, Duffy A, *et al.* TDP-43 gains function due to perturbed autoregulation in a Tardbp knock-in mouse model of ALS-FTD. *Nat Neurosci* 2018; **21**: 552–63.

30 Nishimura AL, Shum C, Scotter EL, *et al.* Allele-specific knockdown of ALS-associated mutant TDP-43 in neural stem cells derived from induced pluripotent stem cells. *PLoS One* 2014; **9**: e91269.

31 Tann JY, Wong L-W, Sajikumar S, Ibáñez CF. Abnormal TDP-43 function impairs activity-dependent BDNF secretion, synaptic plasticity, and cognitive behavior through altered Sortilin splicing. *EMBO J* 2019; **38**: e100989.

32 Huang S-L, Wu L-S, Lee M, *et al.* A robust TDP-43 knock-in mouse model of ALS. *Acta Neuropathol Commun* 2020; **8**: 3.

33 Sleigh JN, Tosolini AP, Gordon D, *et al.* Mice Carrying ALS Mutant TDP-43, but Not Mutant FUS, Display In Vivo Defects in Axonal Transport of Signaling Endosomes. *Cell Rep* 2020; **30**: 3655–62.e2.

34 Ziff OJ, Neeves J, Mitchell J, *et al.* Integrated transcriptome landscape of ALS identifies genome instability linked to TDP-43 pathology. *Nat Commun* 2023; **14**: 2176.

35 Hung C, Patani R. Elevated 4R tau contributes to endolysosomal dysfunction and neurodegeneration in VCP-related frontotemporal dementia. *Brain* 2024; **147**: 970–9.

36 Smethurst P, Risse E, Tyzack GE, *et al.* Distinct responses of neurons and astrocytes to TDP-43 proteinopathy in amyotrophic lateral sclerosis. *Brain* 2020; **143**: 430–40.

37 Hall CE, Yao Z, Choi M, *et al.* Progressive Motor Neuron Pathology and the Role of Astrocytes in a Human Stem Cell Model of VCP-Related ALS. *Cell Rep* 2017; **19**: 1739–49.

38 Altman T, Ionescu A, Ibraheem A, *et al.* Axonal TDP-43 condensates drive neuromuscular junction disruption through inhibition of local synthesis of nuclear encoded mitochondrial proteins. *Nat Commun* 2021; **12**: 6914.

39 Weskamp K, Tank EM, Miguez R, *et al.* Shortened TDP43 isoforms upregulated by neuronal hyperactivity drive TDP43 pathology in ALS. *J Clin Invest* 2020; **130**: 1139–55.

40 Afroz T, Chevalier E, Audrain M, *et al.* Immunotherapy targeting the C-terminal domain of TDP-43 decreases neuropathology and confers neuroprotection in mouse models of ALS/FTD. *Neurobiol Dis* 2023; **179**: 106050.

41 Tamaki Y, Shodai A, Morimura T, *et al.* Elimination of TDP-43 inclusions linked to amyotrophic lateral sclerosis by a misfolding-specific intrabody with dual proteolytic signals. *Sci Rep* 2018; **8**: 6030.

42 Archbold HC, Jackson KL, Arora A, *et al.* TDP43 nuclear export and neurodegeneration in models of amyotrophic lateral sclerosis and frontotemporal dementia. *Sci Rep* 2018; **8**: 4606.

43 McGurk L, Mojsilovic-Petrovic J, Van Deerlin VM, *et al.* Nuclear poly(ADP-ribose) activity is a therapeutic target in amyotrophic lateral sclerosis. *Acta Neuropathol Commun* 2018; **6**: 84.

44 McGurk L, Gomes E, Guo L, *et al.* Poly(ADP-Ribose) Prevents Pathological Phase Separation of TDP-43 by Promoting Liquid Demixing and Stress Granule Localization. *Mol Cell* 2018; **71**: 703–17.e9.

45 Chung CY-S, Shin HR, Berdan CA, *et al.* Covalent targeting of the vacuolar H-ATPase activates autophagy via mTORC1 inhibition. *Nat Chem Biol* 2019; **15**: 776–85.

46 Coyne AN, Baskerville V, Zaepfel BL, *et al.* Nuclear accumulation of CHMP7 initiates nuclear pore complex injury and subsequent TDP-43 dysfunction in sporadic and familial ALS. *Sci Transl Med* 2021; **13**. DOI:10.1126/scitranslmed.abe1923.

47 Oberstadt M, Stieler J, Simpong DL, *et al.* TDP-43 self-interaction is modulated by redox-active compounds Auranofin, Chelerythrine and Riluzole. *Sci Rep* 2018; **8**: 2248.

48 Hans F, Glasebach H, Kahle PJ. Multiple distinct pathways lead to hyperubiquitylated insoluble TDP-43 protein independent of its translocation into stress granules. *J Biol Chem* 2020; **295**: 673–89.

49 Posa D, Martínez-González L, Bartolomé F, *et al.* Recapitulation of Pathological TDP-43 Features in Immortalized Lymphocytes from Sporadic ALS Patients. *Mol Neurobiol* 2019; **56**: 2424–32.

50 Mann JR, Gleixner AM, Mauna JC, *et al.* RNA Binding Antagonizes Neurotoxic Phase Transitions of TDP-43. *Neuron* 2019; **102**: 321–38.e8.

51 Becker LA, Huang B, Bieri G, *et al.* Therapeutic reduction of ataxin-2 extends lifespan and reduces pathology in TDP-43 mice. *Nature* 2017; **544**: 367–71.

52 Zeballos C MA, Moore HJ, Smith TJ, *et al.* Mitigating a TDP-43 proteinopathy by targeting ataxin-2 using RNA-targeting CRISPR effector proteins. *Nat Commun* 2023; **14**:

6492.

53 Hicks DA, Cross LL, Williamson R, Rattray M. Endoplasmic reticulum stress signalling induces casein kinase 1-dependent formation of cytosolic TDP-43 inclusions in motor neuron-like cells. *Neurochem Res* 2020; **45**: 1354–64.

54 Chen Y, Wang H, Ying Z, Gao Q. Ibudilast enhances the clearance of SOD1 and TDP-43 aggregates through TFEB-mediated autophagy and lysosomal biogenesis: The new molecular mechanism of ibudilast and its implication for neuroprotective therapy. *Biochem Biophys Res Commun* 2020; **526**: 231–8.

55 Mompeán M, Ramírez de Mingo D, Hervás R, Fernández-Ramírez MDC, Carrión-Vázquez M, Laurents DV. Molecular mechanism of the inhibition of TDP-43 amyloidogenesis by QBP1. *Arch Biochem Biophys* 2019; **675**: 108113.

56 François-Moutal L, Felemban R, Scott DD, *et al.* Small molecule targeting TDP-43's RNA recognition motifs reduces locomotor defects in a Drosophila model of amyotrophic lateral sclerosis (ALS). *ACS Chem Biol* 2019; **14**: 2006–13.

57 Mollasalehi N, Francois-Moutal L, Scott DD, *et al.* An allosteric modulator of RNA binding targeting the N-terminal domain of TDP-43 yields neuroprotective properties. *ACS Chem Biol* 2020; **15**: 2854–9.

58 Choi K-J, Tsoi PS, Moosa MM, *et al.* A chemical chaperone decouples TDP-43 disordered domain phase separation from fibrillation. *Biochemistry* 2018; **57**: 6822–6.

59 Duan Y, Du A, Gu J, *et al.* PARylation regulates stress granule dynamics, phase separation, and neurotoxicity of disease-related RNA-binding proteins. *Cell Res* 2019; **29**: 233–47.

60 Liu C, Leng B, Li Y, *et al.* Diallyl trisulfide protects motor neurons from the neurotoxic protein TDP-43 via activating lysosomal degradation and the antioxidant response. *Neurochem Res* 2018; **43**: 2304–12.

61 Jo M, Lee S, Kim K, Lee S, Kim SR, Kim H-J. Inhibition of MEK5 suppresses TDP-43 toxicity via the mTOR-independent activation of the autophagy-lysosome pathway. *Biochem Biophys Res Commun* 2019; **513**: 925–32.

62 Wang Y, Liu F-T, Wang Y-X, *et al.* Autophagic Modulation by Trehalose Reduces Accumulation of TDP-43 in a Cell Model of Amyotrophic Lateral Sclerosis via TFEB Activation. *Neurotox Res* 2018; **34**: 109–20.

63 Wilkins OG, Chien MZYJ, Wlaschin JJ, *et al.* Creation of de novo cryptic splicing for ALS and FTD precision medicine. *Science* 2024; **386**: 61–9.

64 Ke YD, van Hummel A, Au C, *et al.* Targeting 14-3-3θ-mediated TDP-43 pathology in amyotrophic lateral sclerosis and frontotemporal dementia mice. *Neuron* 2024; **112**: 1249–64.e8.

65 Audrain M, Egesipe A-L, Tentillier N, *et al.* Targeting amyotrophic lateral sclerosis by neutralizing seeding-competent TDP-43 in CSF. *Brain Commun* 2023; **5**: fcad306.

66 Gao N, Huang Y-P, Chu T-T, *et al.* TDP-43 specific reduction induced by Di-hydrophobic tags conjugated peptides. *Bioorg Chem* 2019; **84**: 254–9.

67 Prasad A, Raju G, Sivalingam V, *et al.* An acridine derivative, [4,5-bis{(N-carboxy methyl imidazolium)methyl}acridine] dibromide, shows anti-TDP-43 aggregation effect in ALS disease models. *Sci Rep* 2016; **6**: 39490.

68 Martínez-González L, Gonzalo-Consuegra C, Gómez-Almería M, *et al.* Tideglusib, a Non-ATP Competitive Inhibitor of GSK-3β as a Drug Candidate for the Treatment of Amyotrophic Lateral Sclerosis. *Int J Mol Sci* 2021; **22**. DOI:10.3390/ijms22168975.

69 Wenqiang C, Lonskaya I, Hebron ML, *et al.* Parkin-mediated reduction of nuclear and soluble TDP-43 reverses behavioral decline in symptomatic mice. *Hum Mol Genet* 2014; **23**: 4960–9.

70 Heyburn L, Hebron ML, Smith J, *et al.* Tyrosine kinase inhibition reverses TDP-43 effects on synaptic protein expression, astrocytic function and amino acid dis-homeostasis. *J Neurochem* 2016; **139**: 610–23.

71 Imamura K, Izumi Y, Watanabe A, *et al.* The Src/c-Abl pathway is a potential therapeutic target in amyotrophic lateral sclerosis. *Sci Transl Med* 2017; **9**. DOI:10.1126/scitranslmed.aaf3962.
